# De l'Auscultation mediate; ou Traité du Diagnostic des Maladies des Poumons et du Cæur, fondé principalement sur le nouveau Moyen d'Exploration

**Published:** 1820-01-01

**Authors:** 


### VIII.

*De l'Auscultation mediate; ou Traité du Diagnostic des Maladies des Poumons et du Cœur, fondé principalement sur le nouveau Moyen d'Exploration.* Par R. T. H. Laennec, Medicin de l'Hôpital Necker, &c. Tomes ii. 8vo. pp. 900. avec Planches. A Paris, 1819.

The heart, the brain, and the lungs have not been inaptly termed by Bordeu, "the tripod of life," since it is certain that neither of these viscera can long bear any extensive lesion, either of function or structure, without endangering existence. Diseases of these organs are the more difficultly distinguished, as the viscera themselves are surrounded by a bony structure, which permits us not to ascertain by pressure the seat and nature of the complaint, as in affections of the abdominal region.

In respect to the chest, the diseases of its contents are very diversified, but not so much so the symptoms which they produce. Cough, dyspnœa, and expectoration are the more prominent phenomena exhibited in organic lesions of the thoracic viscera; and as they attend the most opposite diseases of that quarter, they supply very ambiguous diagnostics. Hence, in all ages, physicians have endeavoured to find out other means of ascertaining affections of the chest than through the medium of the symptoms accompanying them. The idea of applying the ear to the thoracic parietes, and learning by the sound to distinguish different lesions within (the auscultation or listening of Laennec) is not entirely new. Hippocrates, (*Lib. ii. de Morbis,*) advises this process for ascertaining empyema, and the percussion of Owenbrugger and Corvisart are somewhat analogous. For several years past, Dr. Laennec, physician to Necker's Hospital, a man of talent and great industry, has endeavoured to improve the means of diagnosis in pectoral diseases, by what he terms "mediate auscultation," or the transmission of sound from the interior of the chest to the ear of another person, through the medium of a conducting instrument, now to be described. This instrument, which our author denominates by the term "*Stethescope,*" is a cylinder of pretty heavy wood, about a foot in length, and an inch and a half in diameter, pierced longitudinally through its centre by a small hole of a quarter of an inch in diameter, and with one end hollowed out in the shape of a small funnel. A piece of the same kind of wood, with a hole in its centre, is made to fill up this funnel, when necessary, like the tompion of a gun, and then the instrument may be considered as a simple tube. In this last form it is best adapted for ascertaining certain phenomena attending diseases of the heart.

It is well known in Physics, that sound is better conducted by a solid than by a porous body. Thus, if one end of the instrument in question be placed over the region of the heart, and held there lightly but steadily, as you would hold a pen, with the finger and thumb near the said extremity of the tube, and the other end be held firmly to the ear, each stroke of the heart will be heard with the most surprizing distinctness.

When a man, in sound health, speaks or sings, the modulations of his voice, resounding through all parts of the thoracic cavity, produces a kind of vibration of the parietes, which is sensibly felt by the hand placed thereon. When, however, a part of the lungs, by any disease of their structure, ceases to be permeable by the air we breathe, the phenomena abovementioned ceases also, opposite to that part. The same may be said of the extravasation of a fluid between the lung and thoracic parietes; the sonorous vibration is then no longer distinguishable. But much advantage cannot be drawn from this phenomenon, since a variety of circumstances render it equivocal, as fatness of the integuments, &c. We shall, therefore, pass on to another phenomenon still more interesting.

A female patient, 28 years of age, entered the hospital, under M. Laennec, affected with slight bilious fever, and a recent cough, unmarked by any other character than that of a common catarrh. On applying the instrument under the middle of the right clavicle, our author was surprized to find, on the patient's speaking, that the voice appeared to come almost entirely through the perforation in the centre of the stethescope to his ear, by a species of pectriloquism. This phenomenon only took place when the instrument was applied at the spot abovementioned, or within an inch around it.

Unable to account for this curious incident, he applied the instrument to most of the sick in the hospital, and found about twenty among them who presented the same phenomenon in some part or other of the chest. Most of these were in an advanced stage of pulmonary phthisis; and on dissection after death, ulceration of the lungs was found opposite the parts where the pectriloquism took place. This pectriloquism was more distinct in some portions of the chest, *cæteris paribus*, than in others. For instance, the armpits,—back, between the internal border of the shoulder blade and the vertebral canal;—anterior superior part of the breast, near the sternal extremity of the clavicle. When the cylinder is applied to these points, the voice of the patient appears stronger and nearer the listener, than if the naked ear were applied; whereas, in other parts of the chest it is just the reverse. This, of course, is where no disease exists. Where ulceration obtains, the sound will be more distinct in proportion as the ulcerated cavity is near to the surface of the lungs, and vice versâ.

It may readily be supposed, that these discoveries led our author to an extensive investigation of thoracic diseases; and granting, (what we by no means infer,) that the auscultation is a mere phantom, yet the pursuit of it, like the search after hidden riches, has led to a real treasure of important facts and useful information. It is to these facts and observations that our analytical labours shall be particularly directed.*

From the first Article of the second Chapter, on "Tubercles of the Lungs," we are induced to condense some valuable knowledge, though it may not appear entirely original to some of our more learned readers.

What is called ulceration of the lungs, is not the consequence of inflammation and suppuration of the pulmonary tissue, as was long supposed. These excavations are proved by modern pathologists to result from a liquidation or softening down, and evacuation of tuberculous material, the real cause of pulmonary phthisis.

These tubercles are primarily developed in the form of small granules, semi-transparent, grey, sometimes diaphanous, and almost quite transparent, which, as they increase in size, become yellow and opake, at first in the centre, and successively throughout their whole extent. As they enlarge they unite in clusters, and then appear like cheese. It is about this epoch of their development that the pulmonary tissue itself, hitherto sound, begins to assume a more dense and greyish appearance around the tubercles, from the infiltration into it of original or semi-transparent tuberculous matter. Sometimes, indeed, great masses of tuberculous matter form by a

---

* We may here state, however, that a Committee of the INSTITUTE, appointed to examine M. Laennec's Memoir on Auscultation, on the 29th of June last, confirm the leading positions of our author, and pass a high eulogium on his talents and character. The report is signed by Portal, Pelletan, Percy, and Delambre,—all "honourable men"—

similar infiltration, without assuming the miliary or granular modification. The pulmonary structure thus becomes condensed, and impervious to the air; when cut into, the surfaces appear smooth and polished. In whatever way these tubercular accretions are formed originally, they, in process of time, become soft and liquid; this liquefaction commences in the centre of each mass, and gradually approaches the circumference. In this state, the contents of the tubercle present two different forms. Sometimes they resemble thick, yellow, inodorous pus; at others, there are two distinct kinds of substance mixed—one a thin liquid, more or less transparent; the other an opake caseous matter, somewhat soft and friable.

When the tuberculous matter is completely softened down, it opens for itself a passage into one or more of the nearest bronchial tubes; and this opening being narrower than the excavation with which it communicates, they both, of necessity, remain *fistulous* after the evacuation of the tuberculous matter.

It is very rare to find but a single excavation in a lung thus affected. More frequently these cavities are surrounded by tubercles in various stages of progress towards liquidation, which open successively into the principal excavation, forming those anfractuosities which we often find occupying so great a portion of the respiratory organ. Bands or columns of the pulmonary tissue, condensed, and generally injected with tuberculous matter, traverse these excavations, bearing some resemblance to the carneæ columnæ of the ventricles in the heart

In proportion as the tuberculous matter is evacuated, the interior surface of the excavation becomes lined with a false membrane, thin, even, soft, and easily separable, by scraping with the scalpel. Sometimes, however, this false membrane is absent, and the parietes of the excavation are formed of the pulmonary tissue, condensed, red, and infiltrated with tuberculous matter, in different degrees of development.

M. Bayle believed, that this false membrane secreted the matter expectorated by phthisical patients. Our author is of opinion, that the greater portion of the sputum, in these cases, is the product of bronchial secretion, rendered unnaturally copious by the irritation existing in the lungs. He does not, however, assert, that M. Bayle's opinion is entirely groundless.

If the disease continue a long time stationary, there are developed beneath this false membrane, patches of a yellowish white colour, and resembling cartilage in structure, though softer, adhering intimately to the pulmonary tissue. These patches at length unite, and completely line the tuberculous excavation, having no opening but that where the bronchial tube enters. The texture of these cysts is quite analogous to that of cartilage. Their *external* surface is intimately in adhesion with the surrounding parts, and the tuberculous matter, before its liquidation, adheres strongly to their interior surface.

The above is the common development of tubercles, from which there are some deviations or modifications; but, as they are rare, we shall not touch upon them here. Both M. Bayle and M. Laennec agree, in concluding, that the tubercular development cannot be regarded either as an effect or termination of inflammation.—"Les tubercules ne peuvent être regardés comme un effet ou une terminaison de l'inflammation." 31.—It cannot, however, be denied, our author observes, that pneumonic inflammation, acute or chronic, sometimes coincides with the formation of tubercles, and, in subjects predisposed to the disease, may even excite their formation; while, in other cases, the irritation occasioned by their presence may determine inflammation. Nevertheless, a multitude of facts and dissections prove that the development of tubercles is the result of a constitutional disposition; and, in by far the greater number of instances, unpreceded by inflammation, the latter, when it does take place, being generally posterior in date to the former.

We cannot accompany our author through all the chain of facts and arguments adduced in support of the above-mentioned positions; but we may here remark the great coincidence of opinion between Drs. Bayle, Laennec, and Baron, on certain points of pathology in the disease under consideration. Our readers will remember, that Dr. Baron endeavours to trace the formation of pulmonary tubercles to an hydatid origin. M. Laennec shews, that the primary forms of the said tubercles are globular and transparent, or nearly so. This fact may reconcile the opinions of both. In harmony with Dr. Baron's observations, M. Laennec observes, that there is scarcely any organ or structure in the body which is exempt from the ravages of tubercular formations. It is rare, indeed, that their existence is confined to the lungs. In tubercular phthisis, there is almost always a similar affection of the intestinal parietes, particularly of the mucous membrane, where they produce irritation, and ultimately ulceration, occasioning that distressing colliquative diarrhœa, so general in the advanced stages of phthisis pulmonalis. The following is the order of frequency of tubercular disorganizations which M. Laennec has deduced from a great mass of post mortem researches: 1. The bronchial glands and mediastina. 2. The cervical, mesenteric, and other glands of the body. 3. The liver, in which the tubercular accretions arrive at a considerable size, but rarely soften down into a liquid consistence. 4. The prostate gland, in which, on the contrary, the tubercles frequently become fluid, and leave, after their evacuation per urethram, excavations of greater or less extent. These M. Laennec has very often found in the bodies of men who died of phthisis pulmonalis, and where no pain or embarrassment of any kind accompanied the prostatic affection during life. 5. The surfaces of the peritoneum and pleura, where the tubercles generally appear in their globular and semi-transparent state, and too often destroy life, by inducing dropsy before they come to the stage of fluidity. 6. The testicles, spleen, heart, womb, brain, skull, and vertebræ, have all been found involved in the destructive process of tubercular accretion.

In all these situations, as is proved by the observations of Bayle, Laennec, and others, we shall frequently find a simultaneous development of these morbid growths, and that without pain or other symptom of inflammation. The same, indeed, may be said of pulmonary tubercles themselves, which seldom produce any marked alteration in the health, till their size and number begin to interrupt the function of the lungs. In this last way, they may prove fatal before they become fluid, or discharge their contents into the bronchial tubes, and leave excavations behind.*

We are unable to follow our author through his minute directions relative to the application of the stethescope to different parts of the chest, in order to discover whether ulceration exists in the lungs, as evinced by the pectriloquism, or evolution of sound through the parietes of the thorax along the cylinder to the ear of the physician. In more than two hundred dissections of phthisical patients, in whom, during life, he had discovered the pectriloquism, not one failed to present the ulcerated cavities, opposite those points where the evolution of sound was evident.

"Sur plus de deux cents phthisiques que j'ai ouverts, après avoir constaté leur état à l'aide du cylinder, il ne m'est pas arrivé une seule fois de ne pas rencontrer de cavités ulcéreuses dans un point du poumon où j'eusse trouvé la pectoriloquie d'une maniere evidente." 53.

We cannot therefore but hope, that no foolish pride or prejudice may operate against a full prosecution of this curious investigation, on this side of the channel, where, unhappily, the malady in question comes close home to most of our bosoms.

Every one, of even moderate experience, must be aware that there is no certain diagnostic of pulmonary phthisis. We have all seen the cough, dyspnœa, puriform expectoration, hectic fever, emaciation, and, in short, the whole assemblage of symptoms from which Aræteus drew his frightful picture of the disease, concentrated in the same patient; and yet, contrary to all prognosis or hope, a perfect recovery to take place. It will presently be shewn, our author thinks, that such cases, when taken in conjunction with certain facts brought forward below, authorize the belief that *real tubercular phthisis* is sometimes, though rarely, curable, and that too, after the liquidation and discharge of the tubercles, with consequent excavations in the lungs.

"We find, (says Dr. L.) from time to time, among those who have been long affected with chronic catarrh, but who have died of some other disease, anfractuous cavities in the lungs, lined with a semi-cartilaginous membrane, precisely similar to that which lines old tuberculous ulcerations, as described a few pages back. When

* We may here take occasion to remark, that the *absence* of all *external* and ostensible symptoms of inflammation, by no means proves the *non-existence* of that process. How often do we find, on dissection, the most palpable ravages of inflammatory action, where no indication of the same appeared during life. If so, why may not the formation of tubercles be a process of the sanguiferous vessels, analogous to, if not precisely identical with, what we call inflammation?

the history of these patients is traced with care, we find them always refer the origin of their chronic coughs to a severe disease with which they were previously afflicted, and which, on inquiry, presented all the phenomena of pulmonary phthisis, often to such a degree, as rendered their lives despaired of. On the other hand, in patients who had long laboured, say for some years, under pulmonary phthisis, we find, pretty frequently, certain cavities empty, or nearly so, of tuberculous matter, and completely lined by a semi-cartilaginous membrane; while other excavations present a cartilaginous lining of a more soft consistence, incomplete in its extent, and containing a considerable quantity of tuberculous material. The formation of this semi-cartilaginous membrane, then, on the surface of tubercular excavations, appears to be evidently a conservative effort of Nature, and may be looked upon as a kind of internal cicatrix, or rather fistula, occasioning no more inconvenience than some fistulæ of external parts. All those whom I have mentioned in Sect. 94, had died of diseases quite unconnected with phthisis. They had all lived a greater or less number of years in a state of very tolerable health, affected only with chronic cough, or, a few of them, with dyspnœa; but without fever or emaciation."

Our author here introduces a great number of cases in illustration of the foregoing observations, of which we shall condense one or two.

" Case 1. *Pulmonary Ulcerations cured by their Transformation into Semi-cartilaginous Fistulæ.*

" —— Day, a woman about 68 years of age, had been subject to cough and expectoration for many years, with habitual dyspnœa, aggravated by even moderate exercise. With the exception of these symptoms she enjoyed tolerable good health, and attended day and night on an octogenarian lady. Her appetite was good; she was somewhat embonpoint, but her lips and cheeks were of a violet hue.

" On the 31st of December, 1817, she was seized with fever, great difficulty of breathing, cough, and viscid, frothy expectoration, which were a little relieved by venesection. On January the 3d, she was removed to Necker hospital, and being examined with the aid of the cylinder, presented the following symptoms. The respiration could scarcely be heard from any point of the thorax; but a wheezing [râle] was distinctly audible from the inferior and left side of the breast to the fourth rib, or thereabouts. Percussion elicited a confused and heavy sound from the same part. The strokes of the heart communicated no impulse by the cylinder, although they were audible from most parts of the thoracic parietes. The external jugular veins were dilated. The oppression and expectoration were as before described. The following diagnosis was noted at the time. *Inflammation of the inferior part of the left lung; slight dilatation of the ventricular cavities of the heart.*

" Venesection, leeches, blisters, procured but trifling relief, and she sank on the second day after being received.

" *Dissection.* The lungs universally adherent to the costal pleura by bands of ancient date. *Right* lung crepitous and perfectly sound; but presented, at its upper part, an excavation capable of containing a large filbert. The interior of its cavity lined with a polished, thin, and even membrane of a semi-cartilaginous consistence, and mother-of-pearl appearance, into which cavity several bronchial tubes, extremely dilated, opened. The *left* lung presented at its upper part an irregular cavity, the principal excavation of which would contain a nut, and into which a great many brouchial tubes, the size of a crow-quill, were seen to enter; the mucous membrane of these tubes was continuous with that which lined the excavations, and which was similar to the one above described, except being of a somewhat softer consistence. This cavern contained only a small quantity of colourless serosity. No tubercles in the lungs. The pulmonary structure around these sacs was sound, and permeable by the air. The whole inferior part of the left lung was hepatized, from which a puriform fluid, mixed with blood, could be squeezed. The heart somewhat enlarged. The right ventricle passively dilated."

" Case 2. *An Ulcer of the Lungs transformed into a Semi-cartilaginous Fistula, in a Subject presenting another Ulceration not transformed, and several Tubercles in a crude State.*

A woman, 40 years of age, well formed, entered the hospital on the 19th of December, 1817. She stated, that she had long been afflicted with cough and tightness of respiration, aggravated by certain states of the atmosphere. But these complaints had never confined her to bed, till within this fortnight. Examined the day after her arrival in the hospital, she exhibited the following symptoms. She could only keep the sitting posture in her bed. The face was pale and puffed; the eyes sunk and watery; the lips violet; inferior extremities œdematous; breathing short, easily accelerated, and panting. Immediately below the clavicles we could hear, through the medium of the cylinder, a well-marked rattling [râle] in the lungs. The parietes of the thorax were elevated at each inspiration in such a manner as to communicate a disagreeable shock to the ear by the cylinder. The cough produced a yellow and opake expectoration. No *pectriloquism* could be perceived at this time by the aid of the instrument. *The external jugular veins presented a well-marked pulsation.* The strokes of the heart were clearly audible by the cylinder, and were deep and regular. The following diagnosis was noted. *Phthisis without disease of the heart.* Leeches to the epigastrium—pectoral emulsions.

" 21st. The nose and lips livid; respiration short, and hurried; supination impossible; insomnium. This day the strokes of the heart communicated some *impulsion* along the instrument, (la contraction des ventricules donnait quelque impulsion). This symptom, joined with the jugular pulsation, somewhat modified the preceding diagnosis, and led me to suspect that the parietes of the right ventricle of the heart were a little thickened. From the 22d to the 27th, there was a diminution of the livor of the face, and the dyspnœa; the cough being frequent, and the expectoration copious. This amelioration, however, was transient, and early in January, the difficulty of respiration returned with increased violence, while dropsical infiltrations in the lower extremities and left side of the body, made considerable advances. On the 18th, pectriloquism was evident between the fourth and fifth ribs of the right side, a part which had not previously been examined, &c. She died on the 19th of January.

" *Dissection.* The heart was sound in structure, with the exception of, perhaps, a slight thickening of the parietes of the right ventricle. The auricle of this side was distended by partially coagulated black blood; the appendix auriculæ exactly filled with a polypus-like concretion, somewhat fibrinous. About a pint of serum in the left side of the thorax. The lung of this side adherent to the costal pleura, at the uppert part, by a strong band, and at this part were found about a dozen of small tubercles the size of hemp-seed, yellow and opake at the centre, semi-transparent towards the circumference. Here also was found a small excavation lined by a soft and whitish false membrane, which being removed, shewed the pulmonary tissue itself forming the parietes of the cavity, and somewhat red and indurated. This cavity was filled with tuberculous matter, partly softened down into a caseous consistence, partly purulent. The rest of the left lung was crepitous, but its vessels gorged with blood. The whole of the right lung adhered firmly to the costal pleura. At half an inch in length from the intercostal space, where the pectriloquism was heard through the cylinder, an excavation was found capable of holding a nut, lined by a semi-cartilaginous membrane, and containing a small quantity of yellow purulent matter. Into this cavity a dilated bronchial tube opened, but was obstructed by a calcareous concretion."

We are unable to bring forward any more of the cases elucidating the point in question, though many of them are highly interesting. Our author here observes, that although pulmonary phthisis is but seldem cured in this way, yet he thinks the fatal issue is often warded off for a long time by these sanative efforts of Nature. As, however, there is generally a succession of maturating tubercles, the constitution is ultimately broken by the irritation accompanying their expectoration. At all events, the facts here described should prevent us from giving a prognosis decidedly fatal in all cases of pulmonary consumption, since we may be sometimes wrong in so doing, as the following case will shew.

" M. G. an English prisoner of war, 36 years of age, was attacked, in September 1813, with severe hæmoptysis, followed for a time with dry cough, but, at the end of some weeks, a yellow and purulent expectoration. To these were added hectic fever, dyspnœa, and nocturnal perspirations. Emaciation and debility advanced rapidly. The chest sounded well, except under the right clavicle and in the right arm-pit. The hæmoptysis returned from time to time, but in moderation; and in December a colliquative diarrhœa was, with great difficulty, restrained by opiates and absorbents. At this time M. Bayle and M. Hallé saw him in consultation, and considered him as a completely lost case. On the 15th of January Mr. G. experienced a most severe fit of coughing, in which he spat up a mass the size of a nut, which, on examination, proved to be a tubercle, part of it softened down to the consistence of cheese, and other parts into a pulpy and puriform substance. After this event he gradually emerged from the very last stage of phthisis, and ultimately recovered completely."

The Section on the nature of the expectoration in phthisis contains many interesting observations. Our author states correctly, that the expectoration of mucus and phlegm, so long anterior to that of tuberculous matter, shews the irritation occasioned in the aerial passages by the maturating tubercles, and that, on the appearance of the purulent sputum, there is generally a temporary mitigation of all the symptoms. It is at this epoch, of course, that pectriloquism, through the medium of the cylinder, becomes manifest, and serves to accurately distinguish the disease, as depending on a vomica, from those bronchitic affections so closely resembling it in all other respects.

The third Chapter is on a subject hitherto, as far as our reading extends, unnoticed by pathologists; namely, a *morbid dilatation of the bronchial tubes*, found in people who have died after being long subject to chronic catarrh.

" This dilatation is sometimes so considerable that ramifications, which in their natural state would scarcely admit a fine stilet, acquire the diameter of a goose quill, or even that of a finger. These dilated bronchial tubes terminate in cul-de-sacs or cells, capable of holding from a grain of hemp seed to a cherry-stone, filbert, or even an almond. Their mucous membrane is generally red or violet-coloured, and evidently thickened. The cartilaginous rings become blended with the said membrane, and changed into a kind of fibrous tissue. This organic lesion may take place in all parts of the lungs, but is most common in the superior lobes; generally affecting a small, but occasionally a great number of the aerial conduits; in which case, the dilatation is proportionally extreme in the minute ramifications. The bronchial common *trunks* indeed are rarely the seat of this dilatation, even when the sub-divisions acquire a size as great as themselves. When these bronchial dilatations are numerous, the intermediate pulmonary tissue becomes flaccid, and deprived of its natural quantum of atmospheric air.

" The morbid organization in question, owes its origin always to chronic catarrh, or any other complaint which occasions long and violent fits of coughing; hence the whooping-cough is, probably, the most frequent of all causes. When the affection is confined to one or two bronchial tubes, no other inconvenience results than an habitual cough, accompanied by a trifling mucous expectoration; but when it is extended to a considerable number of the minuter ramifications, the consequence is a chronic catarrh which remains for life, and sometimes abridges the range of existence, by the exhaustion and dyspnœa resulting from long and harrassing paroxysms of coughing; but especially from the profuseness of the expectoration, which at length becomes, in all respects, puriform."

*Vol. i. p.* 124, 5, 6, 7.

We may take this opportunity of stating, that we must seldom be considered as translating *literally* from the French. This would not suit the analytical brevity of our style, in which we are somewhat more economical of words than our neighbhouring brethren. We hope, however, that we shall not be forced to cede to others the power of conveying a more faithful representation, even when they employ a much greater number of words for that purpose.*

The morbid dilatation, in question, occasions a degree of pectriloquism, when the cylinder is applied to corresponding parts of the chest, which puzzled our author greatly, and, we apprehend, will puzzle others still more. We shall here introduce to the English reader a sketch of three cases of this curious affection.

" *Case* 1. Master Lajoie, three years and a half old, entered the Hopital des Enfans, on the 30th of January, 1808. He had coughed incessantly, with pertussis, for three months. The paroxysms were only at the distance of some hours, but followed by a fœtid, copious, and, in fact, purulent expectoration, which ran from the mouth. On the 3d of February, the child could only lie on the left side, which, on percussion, emitted a dull sound. A blister was applied to that side. 14th of February, the blister not having produced any good effect, a cautery was applied to the arm. In the intervals of coughing, he complained of no pain; his sleep was tranquil, but the face and hands became puffed, and a diarrhœa came on. The skin was now hot, the pulse small and frequent, the thirst intense. For the last three days the expectoration diminished, and on the 16th, it was suppressed, and death soon closed the scene.

" *Dissection.* No effusion into the serous cavities; the left lung slightly adherent to the costal pleura, and its inferior portion heavy

---

* It is but justice to acknowledge, that M. Laennec's style is much more concentrated than that of his countrymen in general; and is very free from tautology and circumlocution. Our French brethren complain loudly of the style of English medical writers. We do not defend the *latter*; but we cannot help expressing a wish, that the *former* would begin the reformation at home. We would recommend to their consideration, the Fable of the two Crabs, one of which harrangued the other on the aukwardness of her *sideling* gait, without setting any example of direct progression, on her own part. Rev.

and livid. From a longitudinal incision in this part, there issued, on slight pressure, a purulent fœtid liquid, which was contained in a multitude of cylindrical cavities, situated close together, and freely communicating with one another. The largest of these would admit the point of the finger, the others a large pea each. Their communication with the bronchial tubes, of which, in fact, they were prolongations, was distinctly traceable. The mucous membrane lining them was of a deep red and livid colour, but without the slightest breach of structure, so that the puriloid expectoration was, in reality, a secretion. In the parietes of these cavities, the cartilaginous structure was changed to a fibrous, or one of condensed cellular membrane. The conduits above described formed at least three-fourths of the lower portion of lung where they were situated, the intermediate pulmonary tissue being compact, but not indurated, as around tubercles. It was totally impermeable by air, and had lost its cellular structure. The right lung was sound, except at its inferior part, where it was gorged with blood. The mucous membrane of the trachea was of a red livid colour. The liver was very much enlarged. This may be termed a case of acute bronchial dilatation."

In many parts of this Journal, we have conceded to our continental brethren the superiority over ourselves, in minute *pathological* researches; we shall not then be accused of partiality, if we claim precedence in *therapeutics*. We have often been astonished, to perceive a French physician predict before, and demonstrate after death, the ravages of inflammatory disease, and yet stand by prescribing the most inert remedies from a kind of superstitious dread of what he terms *heroic* remedies, and an obstinate attachment to the *medecina expectans*. That many of our countrymen err, by carrying depletion too far, we have often had occasion to remark; but that our continental brethren verge to the opposite extreme, we are equally convinced, by a careful perusal of their writings. We think that it is incumbent on both parties to profit by the errors as well as the merits of each other.

Upon the foregoing case, we may remark, that when inflammatory symptoms supervene on, or accompany puriloid expectoration, we are not to refrain from depletion on this account. It is evident that these inflammatory actions are extending the original mischief, whatever that may be; and, consequently, it is sound practice to check them by active depletion. Great discharges from mucous membranes are kept up by inflammation; and in checking this last, we diminish the former. Nay, what is termed an ulceration of the lungs, is no bar to judicious depletion, when that ulceration is surrounded by, or its irritating discharges are occasioning, contiguous inflammation.

" *Case* 2. Madam M——, 72 years old, had been affected from the age of 16, with a pulmonic complaint, exhibiting the principal phenomena of phthisis pulmonalis. Hæmoptysis, frequently and easily renewed by slight causes; habitual cough, with expectoration, sometimes puriform, sometimes mucous; respiration short, and often very difficult. She experienced remissions, but very rarely intermissions of these symptoms, which, however, did not prevent her generally from her usual occupations of teaching music. When she entered the hospital, her constitutional malady was somewhat aggravated by the addition of a diarrhœa, and a trifling œdema of the legs. Nevertheless, she saw and felt that her terrestrial career was near a close. The œdema of the lower extremities increased, extended to the upper extremities, then to the trunk of the body, with a corresponding augmentation of the dyspnœa. She could only rest in the sitting posture. Finally, she appeared to sink, rather from the dropsical effusions than from the phthisical affection.

"*Dissection.* Universal œdema; lungs adherent to the costal pleura and mediastinum by cellular bands of ancient formation. This connecting medium was infiltrated considerably, and had a gelatinous appearance. On cutting into the superior lobe of the right lung, we found in its interior a great number of roundish cavities, with smooth but red parietes, somewhat similar in appearance to the interior of the cardiac ventricles, or certain fistulous canals. These cavities, some of which contained purulent matter, while others were empty, had attained different sizes. The largest would admit the point of the thumb. These excavations were separated from one another by partitions of condensed pulmonary tissue. They bore no similitude to the cavities left by the melting down of tuberculous masses, as seen in phthisical cadavers. On minute examination, they were found to communicate with, and, in fact, to be continuations of, the bronchi. The parietes of these cavities presented here and there specks of cartilaginous and osseous structure, but they could not be separated into laminæ, being considerably more dense than sound bronchi, after they have ceased to be cartilaginous. No spot of ulceration could be discovered on any part of their internal surfaces, so that the matter they contained was evidently a secretion. Such was the state of almost the whole of the bronchial vessels in the superior lobe of the right lung. The left presented but a few of the above described bronchial dilatations; and the middle and inferior lobes of both lungs none. The mucous membrane of the larynx and trachea was perfectly sound, as were the heart and liver. The womb was enlarged, and the hymen complete."

We consider the foregoing pathological researches as very interesting, and highly deserving of further prosecution. It is only by minute and patient investigations of this kind, that the healing *art* can ever become a *Science.*

From the first chapter of the Second Part of the work under review, we shall select some observations relative to auscultation of the respiration. When the tompion portion of the cylinder is taken out, the instrument forms a kind of funnel; and this is the proper modification for exploring the breathing. When the funnelled extremity is placed steadily on the chest of a healthy man, and the other end is applied to the ear, there will, at each inspiration and expiration, be heard a light, but a very distinct murmur, indicative of the air permeating the cells of the lungs. The same will be heard if the instrument be applied to the larynx or trachea; but the noise is deeper, and somewhat different. Every thing should be perfectly still in the room where this process is tried. The quicker the respiration, the more audible the noise through the cylinder. In infancy it is infinitely more sonorous than in old age.

*Anatomical Characters of Peripneumonia.* In this Section, M. Laennec has displayed great and minute pathological investigation. We shall trace the more prominent features of his delineation of three well defined modifications of inflammation in the respiratory organ.

*First Modification.* The lung, more heavy and firm than natural, presents exteriorly a livid or violet colour, but is still somewhat crepitous, though, when pressed between the fingers, it gives the feeling of engorgement. When cut into, its tissues appear of a red livid hue, and injected with a serosity more or less bloody and frothy, which flows abundantly from the cut surfaces. The spongy or cellular texture of the organ, however, is still discernible.

*Second Modification.* Here the crepitus is gone, and the lung acquires a density and weight analogous to liver; hence the term *hepatization*, which is sufficiently expressive and correct. From the cut surfaces of such a lung, there is scarcely any exudation; but, on scraping them with the scalpel, one collects a small quantity of fluid, composed of serum, blood, and frequently some puriform matter. On examining these cut surfaces, by aid of a strong light, we can discover no cellular structure, but rather a *granulated* surface, which *granulation* appears to our author to be the proper anatomical character of pulmonic *inflammation*, in contra-distinction to *tubercular* engorgements, and many other indurations to which the lung is subject. When a lung is entirely hepatized, it appears, on opening the thorax, to be enlarged in volume; but this is a deception arising from its not shrinking, like a healthy lung, on exposure to the air.

*Third Modification.* Here the pulmonary tissue, while it preserves the same density and granulated aspect, before described, assumes a pale yellow or straw colour, and exudes, more or less plentifully from its cut surfaces, a yellow, opake, viscid, and purulent matter, not so disagreeable in smell as the pus from an external wound.

"This is, properly speaking, a pulmonary suppuration, for, notwithstanding the opinions both of ancient and modern physicians, relative to those purulent depôts termed vomicæ, I affirm that there is not a more rare organic lesion of the respiratory apparatus, than a real collection of pus in the pulmonary structure."

Our readers are aware that M. Laennec has endeavoured to prove, a few pages back, that what are supposed to be suppurations or vomicæ, are merely a softening down of a tuberculous mass, not necessarily accompanied, nor caused by any inflammatory action. Out of several hundred dissections of people who had died of pulmonic inflammation, our author did not meet with more than five or six instances of suppuration, that is, of abscesses in the lungs; and in all these cases, except one, the abscesses were very small, and accompanied by the third modification or degree of inflammation just described. M. Laennec considers that, in general, the function of the organ, and consequently life, is destroyed before an abscess can be properly formed. These three stages or modifications may often be seen in different parts of the same organ, and are otherwise occasionally intermixed; but still sufficiently distinguishable.

Our author thinks, that even the third degree, or puriform infiltration, may terminate in resolution, or rather absorption of the pus, and without further disorganization of the lung. Chronic peripneumony, when uncombined with the development of tubercles, presents precisely the same anatomical characters as the acute.

Our author considers that each symptom, characteristic of pulmonic inflammation, is occasionally absent; and therefore none of them are truly pathognomonic of the disease, if we except the yellowish white, viscid, and semi-transparent sputum, mixed with bubbles of air, which almost invariably appears, in greater or less quantity, in the early days of the inflammation.

*Percussion* of the chest offers a more certain diagnosis than the symptoms; but a great many circumstances occasionally combine to render it inapplicable or uncertain. Investigation by the cylinder, M. Laennec observes, is not attended with these inconveniences. It indicates the pulmonary engorgement under all circumstances, and even the degree of that engorgement, with more accuracy than percussion.

In the *first modification*, a respiration can still be heard at the point affected, but not near so sonorously as in other points, where there is no corresponding disease. Inspiration is also accompanied by a kind of crepitation, or slight rattling,* which may be considered the pathognomonic sign of pneumonic inflammation of the *first degree*.

In the *second modification*, there is a total absence of all respiratory murmur through the cylinder, unless catarrh be joined with pneumonia, when there will sometimes be heard a kind of mucous wheezing, instead of the respiratory noise. The cylinder also marks, with great exactness, the progress to recovery. The sound returns in proportion to the renewal of healthy function in the lung. *Percussion*, however, should never be neglected, in aid or corroboration of the cylinder and the external phenomena.

---

* "Râle," throughout M. Laennec's work, signifies what, in this country, is called the "rattles;" the highest degree of which, near death, is familiar to nurses, and denominated the "dead rattles." It is caused, of course, by the mucus accumulating in the air passages, which the patient is unable to expectorate. Rev.

*Emphysema of the Lungs.* This is a disease which has never been accurately described.* In it the air cells become dilated in size, sometimes to that of a cherry-stone, and irregular in shape. When they are large, they are probably owing sometimes to a rupture of several cells; but, unquestionably, a single cell may become dilated to the dimensions above stated. As long as the air is confined to these bags, there is not much inconvenience produced; but when they become distended too far or too suddenly, they burst occasionally, and the air is extravasated into the circumambient cellular structure of the lungs, precisely in the same way as it becomes extravasated into the subcutaneous tissue in common emphysema.

The symptoms of this disease are rather equivocal, and we are unable to detail M. Laennec's directions for ascertaining it by percussion and auscultation; but we shall sketch one or two cases, which will be found interesting also in other points of view.

"*Case of Emphysema of the Lungs, with Hypertrophy (active Enlargement) of the Heart, succeeding habitual Catarrh.*

"Mary C——, 41 years of age, born of healthy mother, but asthmatic father. Having experienced a severe illness at the age of four years, she was ever afterwards subject to head-aches, cold feet, and winter catarrh, accompanied by frequent bleedings from the nose, which, in summer, changed into simply an habitual cough. At the age of eighteen she suffered a severe mental affliction, with great increase of the catarrhal affection. From this period she began to feel great dyspnœa at night, with increase of cough on walking, and frequently vomiting. At thirty-five she married, and during the first pregnancy the head-aches and epistaxis disappeared, but the dyspnœa was aggravated. At thirty-eight she lost her husband, and experienced great reverses of fortune. Soon after this, the menses disappeared, and were succeeded by habitual whites, and augmentation of all the other symptoms. At the age of forty she took, by the advice of an Empiric, some drastic purgatives. In a few days the whites disappeared, as also the head-aches; but the urine now became scanty, and the lower extremities œdematous. Her respiration became so embarrassed, especially at night, that she felt as if the chest were compressed between two boards. The paroxysms of coughing were also extremely distressing, till a slight morning expectoration of whitish frothy sputum ameliorated her condition for a few hours.

"She was admitted into the Clinique Interne of the Ecole de Medicine, on the 27th of February 1802, presenting the following symptoms. Face puffed, œdematous; no head-ache; appetite good; extreme desire to eat charcoal; respiration short and difficult; severe paroxysms of coughing, especially in the night, followed by mucous expectoration in the morning, with some relief. The chest

* Dr. Baillie takes some notice of this appearance, but gives no account of the symptoms during life. REV.

sounded well every where except under the left mamma. The pulsations of the heart were strong, hard, and embarrassed; the pulse regular, but hard. For three months past, she has had palpitations of the heart three or four times a day; the stomach swells much after eating, as also the abdomen; and then the dyspnœa is distressing. There is an obscure feel of fluctuation in the abdomen, with infiltration of the lower extremities. She starts from sleep. Corvisart pronounced the following diagnosis. "*Organic disease of the heart from original and habitual debility of the lungs.*"* She died on the 15th of March following.

*Dissection.* Left side of the thorax sounded much more dully than the right; the latter appeared bulged out more than the left side. The right lung much more voluminous than natural. It did not shrink on exposure, and was emphysematous. There were a few crude tubercles here and there, but no effusion. This lung presented, on its surface, vesicles filled with air, formed by elevations of the pleura, and most of them as large as a pigeon's egg; but some of them smaller. The left lung, though less voluminous, was in a similar state as the right, except that the emphysema was less strongly marked. The pericardium contained no fluid. The heart was greatly enlarged; the parietes of the left ventricle an inch in thickness, and very firm. Some effusion in the abdomen, but no disease of its viscera."

CASE 2. *Total Emphysema of the Lungs.*

J. B. Cochard, 37 years of age, entered Necker Hospital on the 25th of May, 1818, for the treatment of œdema of the lower extremitities, of some days' standing. He stated that he was affected with habitual cough and mucous expectoration from the third year of his age, and which he attributed to a cold damp cellar in which he was nursed. His respiration was also embarrassed for many years past. He now presented the following symptoms, on examination. Lips bluish; respiration short and embarrassed; cough frequent, followed by a ropy expectoration; pulse frequent, but regu-

---

* It does not appear that M. Laennec has fallen into the opinion of M. Rostan, who, from a series of cases and observations, imperfectly described and hastily drawn, endeavours to trace asthma, even in its periodical or spasmodic form, to organic lesions of the heart. In M. Ducamp,† who has lately translated into the French language, the able Treatise on Asthma of our distinguished countryman Dr. Bree, M. Rostan has found a close reasoner and observer, who is likely to subvert this melancholy doctrine which, at once, destroys all hope in the mind of either patient or physician. M. Rostan's Pathology of Asthma, indeed, is incompatible with the symptoms, treatment, and *post mortem* appearances, recorded by the most candid and accurate observers in ancient and modern times. The etiology and symptoms of asthma are too various to admit of this exclusive system of causation.

† See Journal General de Medicine for October, 1819.

lar; infiltration of the abdominal integuments, scrotum, and lower limbs. The thorax sounded well every where; but on application of the cylinder, the respiration could not be heard any where, even when the patient breathed with all his efforts. The beating of the heart gave little impulse or sound along the cylinder. After various vicissitudes of being better and worse, but still presenting the foregoing phenomena, he terminated his career on the 19th of October following. It may be observed here, that M. Laennec, in the presence of Cayol and others, predicted emphysema of the lungs, and noted it on the books, long before the patient's death. M. Cayol assisted at the examination, having attested the phenomena developed by the cylinder, as above stated.

*Dissection.* Meninges of the brain somewhat injected; but no effusion in the ventricles. The heart double its natural size; the left ventricle of great dimensions, and the parietes thickened, red, and firm; the right ventricle of still greater volume, and its parietes unnaturally thickened.* No adhesion of the lungs to the internal surface of the chest; they did not shrink. On many parts of the surface were seen transparent vesicles evidently formed by an elevation of the pleura, and distended with air. Their size varied from that of a filbert to that of an almond, or larger. The specific gravity of the lungs was one half that of a healthy lung. Thrown into a pail of water, they floated on the surface with only a few lines of dip. When squeezed between the fingers, one perceived the displacement of an elastic fluid rather than a natural crepitation of the pulmonary tissue. When cut into, there was a slight extrication, or rather explosion of gas. The whole of the substance of the lungs was emphysematous, excepting a few points towards the root of the organ; from the cut surfaces of which trickled a frothy and sanguineo-serous fluid. In other respects the lungs were healthy. The liver was gorged with blood, and the mucous membrane of the stomach and intestines was of a deep red colour."

We are unable to introduce any more of these cases of pulmonary emphysema, though very many are related by M. Laennec. We must also pass over entirely the third chapter, on "Accidental productions developed in the lungs," though we recommend an attentive perusal of this chapter to those who are acquainted with the language, and have access to the original work.

At page 324 of vol. I. we have the following observations. When crude tubercles, of no great size, are equally dispersed through the lungs, the respiration gives no indication of them through the cylinder; nor can any thing be learnt from percussion of the chest. But if the tubercles be irregularly distributed in masses of some size, in any part of the lungs, then at the corresponding points of

---

* This patient offers an example of active enlargement of the heart, which presented no sign of its existence by the cylinder, percussion, or general symptoms. When we come to diseases of the heart, we shall shew the cause of this absence of signs in some particular cases." 245.

the thorax, no respiratory sound will be audible through the *stetho-scope*; and, on percussion, the said points will emit a dull sound, different from what is emitted from a healthy chest, or from other points of the same chest. Thus crude tubercles present phenomena through the medium of the cylinder just the reverse of what tuberculous excavations, or as they are termed, ulcerations do.

The fifth Chapter of the first volume is on Pleurisy, and is a very valuable pathological article. We shall use our endeavours to convey to the English readers as much as possible of its contents, in our own language and idiom.

Our author asserts, that inflammation of the parenchymatous structure of the lungs, and of their covering or pleura, may, and do exist separately. It much more frequently happens, he thinks, that peripneumonia is combined with pleuritis, than pleuritis with peripneumony. The anatomical character of acute pleuritis is *redness* of the tissue, with a greater development than natural of the blood-vessels, as though they were injected.

" Some physicians (says M. Laennec) consider that a *thickening* of the pleura is a common result of inflammation there; but this character has never appeared evident to me. It is certain, that in the greater number of cases where this supposed thickening was found, a *layer of tubercles* developed on the internal or external surface of the tissue,—cartilaginous incrustations—or false membranes, more or less dense, and intimately adherent to the membrane, were mistaken for an increased thickness of the said tissue." 330.

Our readers will not fail to perceive that M. Laennec's pathological researches have made him familiar with the morbid structure described by Dr. Baron, in his valuable work on " Tuberculated accretions of the serous membranes."

Inflammation of the pleura is always accompanied by an exhalation from its internal surface; which, in fact, is the suppurative process of serous tissues, and commences with the inflammation. This exudation presents two different substances; one demi-concrete, the other quite aqueous. The first is termed *false membrane*, the other *serosity*, or sero-purulent effusion. The thickness of these false membranes varies from half a line to two lines; and they are, in general, of pretty uniform dimensions, but sometimes not. When the serous effusion is considerable, the false membranes are often detached, and float about in the liquid. The serous effusion almost invariably accompanying the formation of false membranes, is generally of a yellowish or orange colour, the transparency of which is only troubled by floating fragments of the concretion above described. In some cases, the effusion has a sanguineous tinge, or is even entirely bloody. In proportion to the strength of the inflammation is the relative proportion of false membrane to the serous effusion. On the contrary, in feeble and debilitated subjects, the effusion predominates relatively over the albuminous concretions, " and in this way pleuritic inflammation blends insensibly with hydrothorax." 334.

If Nature be not disturbed in her course, and the disease do not proceed violently to a degree beyond her sanative powers, the serosity becomes absorbed, and the false membrane organized, forming those adhesions which we so commonly meet with between the pulmonary and costal expansions of the pleura.

The anatomical characters of *chronic* pleurisy do not differ essentially from those of the *acute*. The effused fluid, in the former case, is not, in general, so limpid or inodorous as in the latter. Chronic pleurisy seldom tends to a spontaneous cure; nor does nature seem able to arrest the progress of the effusion, or convert the false membranes into cellular tissue. A cure, however, sometimes takes place in a way which shall be presently described.

In chronic pleurisy, the effusion commonly increases from day to day. The affected side swells out; the intercostal spaces enlarge, and become on a level with, or even rise higher than, the ribs. The lung pressed up against the mediastinum or vertebral column, and retained by the pseudo-membranous exudation with which it becomes coated, is sometimes reduced to the smallest possible volume, and rendered impermeable to the air. This is the empyema of modern authors; for few pathological physicians will now assert that empyema is produced by a vomica opening between the two pleuræ instead of into the bronchiæ. A maturated tubercle will sometimes open into the cavity, so called, of the thorax, and there cause considerable effusion, by exciting chronic pleurisy, and its consequent serous exudation. Chronic pleurisy never, at any epoch of its course, presents the fever, sharp pains, and reaction which characterize the acute species. It generally attacks those whose constitutions have been broken down by any cause; and especially those whose lungs are kept in irritation by scrofulous tubercles.

The *symptoms* of acute pleuritis are generally pretty well marked, though not always unequivocally so; but chronic pleurisy is often so faintly characterised, and accompanied by so many anomalies in the different functions, that it is not, for the most part, till after many weeks or months, that the real nature of the disease is suspected. In such cases, percussion and auscultation offer surer means of diagnosis than the symptomatic phenomena. From the moment that *effusion* takes place, percussion elicits no sound from the corresponding thoracic parietes. Auscultation is still more unequivocal. When the effusion is pretty considerable, there is a total absence of all respiratory sound through the cylinder, from any part of the thorax, except along the vertebral canal. This cessation of sound, soon after the commencement of acute pleurisy, is a pathognomonic sign that an abundant effusion has taken place; for when, in peripneumonic inflammation, the sound also disappears, it is always very gradual, and at the same time partial, the upper portions of lung not ceasing to emit sound for many days, or even weeks, after the commencement of the disease.

"In considerable pleuritic effusions, the respiration will occasionally be heard through the cylinder just *under the clavicles*, as well as along the vertebral column, in which last place it is always audible. In these cases, we may be sure that there are adhesions of ancient date between the pulmonary and costal pleuræ at the subclavicular points abovementioned." 349.

When in old people and cachectic adults labouring under pleurisy, the cessation of respiratory sound is sudden and complete, the prognosis ought, in general, to be unfavourable; because we may be certain that Nature will not be able to organize the false membranes, or absorb the effused fluid, consequently the pleurisy will become chronic, and the accumulation increase. In children, and people of good constitution, the effusion is seldom considerable; and the respiratory sound returns in the side affected, after a few hours or even a few days suppression of it, but not to the same degree as in the sound side.

If we strip a pleuritic patient, in whom considerable effusion has taken place, we shall generally perceive a dilatation or bulging out of the affected side, as has often indeed been remarked by ancient as well as modern physicians; but a tightening or contraction of one side of the thorax, together with a want of respiratory sound, remaining after a pleuritic attack and the absorption of an effused fluid, have not been hitherto much noticed, or accurately described by pathologists. If we measure with a cord, from the centre of the sternum round to the spine, we shall often find more than an inch difference in the contours of the two sides. The contracted side of the thorax is also shortened. The shoulder is lower, and the pectoral muscle smaller than on the opposite side. But this inequality is more perceptible by the eye than by admeasurement. The vertebral column usually preserves its perpendicularity, but the patient walks like one who is a little lame.

"M. Laennec has found by dissection, that this lateral contraction of the chest is owing to a kind of irregular termination of chronic pleurisy, in which the sero-purulent effusion having continued a long time, the false membranes covering the pleura and lung had acquired a firmness, resembling the rind of bacon, which prevented its subsequent conversion into cellular tissue. When at length the effused fluid was absorbed, the lung so long compressed by it, remained bound down by the rigid false membrane alluded to, and was incapable of expansion in proportion to the absorption; the consequence of which was, that the ribs of that side fell in a little, and the whole appeared contracted."

*Hydrothorax.* M. Laennec considers *idiopathic* dropsy of the pleura as an extremely rare disease. He has not found it in a greater proportion than about one in two thousand dead bodies. It has too often been considered *idiopathic* by superficial pathologists, when it was the *effect* of enlargements of the heart, aneurisms of the aorta, or even organic affections of some of the abdominal viscera, especially the liver. It has also, in no small number of cases, been confounded with the effusion of pleuritis, when that effusion happened to be unusually transparent. The disease when idiopathic, is generally confined to one side. Its anatomical character consists simply in an accumulation of serosity in the bag of the pleura, without any visible alteration in the texture of that tissue. The lung, of course, is compressed up against the mediastinum, and sometimes almost annihilated. The most constant *symptom* is a tightness in the respiration. Percussion elicits a dull sound, and there is no evidence of the respiratory movement in any part of that side of the thorax, except opposite the root of the lung. Nothing but the general history of the case can enable us to distinguish idiopathic dropsy of the pleura from the inflammatory effusion of the same. And, in short, whatever difference there may be in the symptoms and post mortem appearances of dropsy from sheer debility, or the lesion of an important organ as the heart, and serous effusion from inflammation of the expanded tissues, they are very frequently blended together at some period of their march. In what are called common idiopathic dropsies of the abdomen and chest, how frequently do we find semi-transparent albuminous filaments concreted almost to the consistence of the false membranes above described, and undoubtedly the products of inflammatory action.

These facts may explain the reason why some authors have admitted of inflammatory dropsies, and why blood-letting is sometimes useful in diseases of this class. They shew, however, that the same measure is not to be indiscriminately employed in dropsy, since the disease often arises without any inflammatory action in the membranes where the fluid is effused.

*Symptomatic Hydrothorax.* This is as common as the idiopathic species is rare. It is complicated with, or the result of almost every disease, acute or chronic, general or local. It is too often a prelude to the fatal termination; and is most commonly met with in subjects who have died of acute fevers, diseases of the heart, and tuberculous or other diseases of the lungs and different organs,

Hydrothorax also arises from organic diseases of the pleura itself. These are principally *cancerous* and *tuberculous* productions on its surface. The *former* are of the cerebriform kind. They present themselves usually in masses of various sizes, but seldom exceeding that of an almond. They adhere firmly to the pleura, which has a reddened appearance for some way round their bases. They are seldom in great numbers.

" The *tuberculous* productions, on the other hand, developed on the surface of the pleura, are exceedingly numerous, and vary in size from that of a millet seed upwards. They are set close together, and are often united by a false membrane, in which they appear to be studded.

" At an early period of their progress they may be scraped off, in a great measure, together with the said false membrane, which appears their nidus rather than the pleura." ' Qui paraissent evidemment developpés dans son épaisseur et font corps avec elle plutôt qu'avec la plèvre.' 416.

" At a more advanced stage, the false membrane disappears; that is, it becomes organized, and united with the pleura, which is then thickened. The tubercles are now extremely adherent to the pleura, and appear implanted in its substance. Sometimes these tubercles are demi-transparent, greyish, or nearly colourless; at other times, yellow or opake. I have never seen them in a state of maturation, as in the lungs of advanced phthisical patients. The interstices between them are often reddened, and traversed distinctly by blood-vessels. At this period the pleura presents an aspect analogous to that of the skin, affected with certain eruptions. This morbid granulation is not near so common on the pleura as on the peritoneum." 417.

Our Readers will perceive the perfect coincidence and mutual corroboration between Dr. Baron's observations and the foregoing extract, which shews how familiar M. Laennec is with the morbid organization forming the subject of Dr. Baron's work. This article altogether will teach our countrymen the importance of attending to the pathological investigation of our continental brethren, who have superior means of prosecuting this importaut branch of medical science; and, we fear, a greater zeal in the pusuit of it, than ourselves.

The tubercular disorganizations of the pleura, above described, too often give origin to serous effusion of a reddish or bloody appearance. The cylinder will detect the effusion, but not the tubercular disorganization that occasioned it; these must be ascertained by the general symptoms of the disease.

√ *Œdema of the Lungs.* This is a serous infiltration into the cellular texture of the lungs, amounting to a degree that considerably diminishes their permeability to the air. It is a disease very common, but very little known. It occurs most frequently in cachectic subjects, towards the termination of long-continued fevers or organic diseases; but particularly during the convalescence from peripneumonic inflammation. Chronic catarrh also strongly predisposes to this disease, which often terminates their existence by suffocation. The suffocative orthopnoea which carries off children suddenly at the close of measles, is probably no other than œdema of the lungs. It, for the most, occurs as the fatal close of other diseases, a few days or hours before death; but it sometimes continues for many weeks or months. Its anatomical characters are the following.

"The blood-vessels appear to contain less blood than in a healthy lung. The tissue itself is denser and heavier than natural, and does not shrink when the thorax is opened. When cut into, there oozes from the surfaces an abundance of clear or slightly yellow serosity, very little frothy. When there has been an inflammatory condition of the lung, the effusion is of a redder colour and more frothy. The texture of the air cells suffers no alteration. The symptoms during life are very equivocal. Want of freedom in respiration, a slight cough, and a thin watery expectoration, more or less abundant, are the only symptoms indicative of the disease, and these, of course, are common in many other affections of the chest. Percussion affords us no diagnostic assistance in these cases; the cylinder is more satisfactory. The respiratory sound is very imperfectly heard, principally on account of the efforts which the patient is obliged to make in breathing. A slight crepitation or wheezing is heard through the cylinder, the same as in the first degree of peripneumony, from which, in truth, it is very difficult to distinguish the pulmonary œdema, and, therefore, the general symptoms and history of the case must be taken into consideration."

*Vol. ii. p.* 13,14.

We shall only be able to introduce one of M. Laennec's cases in illustration of the disease under consideration; and, that condensed as usual.

" *Case of Œdema of the Lungs supervening on Convalescence from Peripneumony.*

" Mary Basset, a chambermaid, ætat. 40, of variable state of health, had been subject, from youth, to difficulty of breathing and frequent palpitations. She had epigastric pulsation; did not begin to menstruate till 19, and found no benefit from the change. Married at 24, she had three children, after which the menses disappeared. She now became dropsical, for the removal of which complaint a Charlatan administered medicines which produced a great flow of urine that removed the dropsical swellings. The dyspnœa and palpitations were now more supportable; the menses re-appeared. In January 1817, having been up several nights in attendance on a sick person, she fell into a state of great debility, and was threatened with immediate suffocation on moving a little quickly, or ascending a flight of stairs, which last soon became impracticable. She had slight cough, with mucous expectoration; no appetite or sleep. In this state she entered the Hospital Necker on the 7th of March. On examination, she appeared to be labouring under peripneumony, the reigning epidemic. The face was very pale, and slightly puffed; lips blue; lower extremities œdematous; great oppression; frequent palpitations; sleep interrupted by dreaming and starting; throbbings in the head; the beating of the heart communicated no propulsion to the cylinder, but had a clear sound. Several parts of the chest sounded badly on percussion, and emitted no respiratory noise through the cylinder. The following diagnosis was written on her ticket. " *Partial peripneumony of both lungs; passive dilatation of the heart.*" She dragged out a miserable existence till the 2d of June, when she died.

" *Dissection.* General anasarca; skin extremely pale; lips of a violet colour; brain and membranes natural; a trifling serosity in the lateral ventricles; right lung partially adherent to the costal pleura by very soft cellular bands. Half-a-pint [French] of yellow serum in the cavity of the pleura on this side. The anterior part of this lung was sound, but infiltrated with a colourless serosity. The middle and inferior lobes were more compact, and from an incison in them flowed abundance of transparent serum, mixed with a small quantity of a yellow puriform fluid. The left lung was nearly similar, and on this side, about the same quantity of effusion. In the pericardium two ounces of fluid. The heart was somewhat enlarged, and its substance soft, flaccid, and easily torn. The cavities greatly dilated, their parietes exceedingly attenuated. The other viscera sound." *Vol. ii.* 35, 6, 7.

We shall wind up this long, and we trust, important analysis, by introducing to the notice of the English reader, M. Laennec's observations on a subject, the name of which is not very familiar to his ear.

*Pulmonary Apoplexy.* The pathological characters of this disease are very little known; but not so its principal symptom; namely, hæmoptysis. The ancients attributed this alarming occurrence to a rupture of a blood-vessel; and so do many of those physicians of the present day, who think it derogotary to listen to any recent pathological discovery or doctrine, till it is forced upon them by the universality of its adoption, when they commonly admit it without examination.

Modern anatomists have, for some time, known that the above supposed cause of hæmoptysis was imaginary. Hæmoptysis of this kind can only happen in the two following cases; first, where an aneurism bursts into one of the bronchi; secondly, where a blood-vessel gives way in traversing a tuberculous excavation, a circumstance exceedingly rare. These two species of hæmoptysis are quickly fatal, and, consequently, are of a totally different nature from those numerous cases of hæmoptysis which we every day meet, attended or not by fatal consequences. We attribute, with justice, those slight hæmoptyses which accompany catarrhal and pulmonic inflammation, to a disturbance in the vital properties of the mucous membrane of the bronchi, in consequence of which, the said membrane exhales blood, instead of secreting mucus. The same thing appears to result from the irritation of developing tubercles in the pulmonary structure. But those violent and profuse hæmorrhages from the lungs, which are with difficulty restrained by bleeding and other means—nay, which sometimes baffle all our endeavours, depend on a much more serious cause, the first *effect* of which is to produce a profound alteration of structure in the pulmonary texture. This change consists of an induration equal to that of a strongly hepatised lung—(endurcissement égal a celui du pumon le plus fortement hepatisé)—but, in other respects, very different from common hepatisation. This induration is always partial, and never occupies a great portion of lung; its extent being seldom more than from one to four square inches. It is very exactly circumscribed; for at the very point where the induration ceases, the engorgement is equally great as at its centre. The surrounding pulmonary tissue is perfectly healthy and crepitous, offering nothing analogous to that progressively decreasing density of structure which is observed as you cut from the centre of an inflamed portion of lung. The pulmonary tissue indeed is often even pale around the hæmoptysic engorgement; though occasionally somewhat reddened and injected with arterial blood. Yet, in these latter cases, the line of demarcation between the dense engorgement and lighter sanguineous injection is always clearly marked and defined.

The engorged portion is of a very dark red colour, similar to that of a clot of venous blood. When an incision is made into it, the cut surfaces present an appearance of granulation, as in hepatisation; but, in other respects, the aspect of these two alterations of structure is altogether different. In the hæmoptysic engorgement, the indurated portion appears perfectly homogeneous, as we can perceive nothing of the natural texture, except the bronchi and largest blood-vessels, the tunics of which, also, have lost their white colour, and become tinged with blood. If we scrape the cut surfaces with our scalpel, we only procure a small quantity of dark and half-coagulated blood. Sometimes the centre of an hæmoptysic engorgement is broken down and occupied by a clot of pure blood.

"The lesion, under consideration, is evidently the result of a sanguineous exhalation into the parenchymatous structure; that is, into the air cells of the lungs, resembling, in all respects, the cerebro-sanguineous exhalation producing apoplexy, and, hence the term by which I have designated the disease.

"The brain and lungs are not the only organs subject to this species of sanguineous effusion. I have seen it form spontaneously, and, as it were, in the twinkling of an eye, in the subcutaneous cellular tissue; and I have found the same in almost every part of the body; between the coats of the intestines, among the muscular fibres of the heart, and under the cellular envelopes of the kidnies and pancreas. I assisted my colleague, M. Royer-Collard, a few years ago, at the dissection of a man who had died suddenly of apoplexy, in whom we found these sanguineous effusions throughout the cellular tissues of all the members, the trunk of the body, and even in the envelopes of most of the abdominal viscera. Examples are occasionally seen of sudden death from these effusions of blood into the parenchymatous structure of the lungs, and where, on dissection, we find a sanguineous clot in the middle of a lobe, lacerated like the brain in certain cases of apoplexy. The hæmoptysic engorgement described above, is only a less intense degree of the same affection; and where the exhaled blood concretes, as it were, in the air cells, and combining, in some measure, with the pulmonary tissue, through the vital influence, in a manner very different from mere coagulation of blood, out of its proper vessels."

*Vol. ii. p.* 43, 4.

The above species of engorgement is most frequently found towards the centre of the inferior, or the posterior portion of the middle lobe; and, consequently, it is near the spine, and lower region of the chest, that we are to seek for it with the cylinder. It is easily distinguishable, from those *post mortem* infiltrations of blood, which we meet with in the dead body. These last are always liquid, and formed by a mixture of blood and serosity, often frothy, which distils abundantly from the cut surfaces. This kind of engorgement is never circumscribed; but, subject to the common laws of gravity, it is most concentrated in the lowest portion of lung, and gradually diminishes from below upwards.

However intense this affection may be, the resolution of the pulmonary engorgement appears to be readily effected by the powers of Nature, when the hæmorrhage is over; as is proved by the dissections of those who have died of other diseases, after having experienced severe attacks of hæmoptysis. We shall not state the symptoms of the disease, since they are well known. The hæmoptysic engorgement which gives origin to the flow of blood, is of too small extent to be ascertained by percussion; but our author thinks that the cylinder will often detect it. If then, when the precursory symptoms of hæmoptysis are present, and the cylinder shows circumscribed portions of the chest where no respiratory noise can be heard, but only a mucous rattling or wheezing, we may suspect that a flow of blood is at hand, and should consequently take precautionary measures against it. We shall conclude with the following interesting narrative.

*Case in illustration.* " J. Dirichard, an artisan, 45 years of age, had been, for many years, subject to a difficulty of breathing, of a suffocating kind, whenever he attempted to use any violent exercise. When he entered Necker Hospital, in August 1818, he had experienced a constant and distressing dyspnœa for about a fortnight. On the day of his entry he presented the following symptoms: Face pale; pulse scarcely perceptible at the wrist; feet and legs œdematous; no appetite; thirst moderate; sleep short, and broken by startings and dreams. The respiration, though short and confined, could be heard clearly through the cylinder. The chest sounded well, on percussion, every where, except at the region of the heart. The beating of the heart, investigated by means of the cylinder, gave the following results. Impulsion of the left ventricle very strong and sonorous; sound and impulsion of the right ventricle much less so; no action of the auricles could be heard. The following diagnosis was noted on the books. *Active Enlargement of the Heart*, (Hypertrophie du cœur.)

Blood-letting, aperients, regimen, &c. In about a month, the man finding himself well enough to return to his business, demanded his discharge, which was granted. Six weeks afterwards he returned to the hospital, exhibiting precisely the same symptoms which he had done before. The œdema had now reached the integuments of the abdomen; the respiration was permanently tight, yet its passage through the lungs was distinctly audible by the cylinder. General and local blood-letting, aperients, blisters, and digitalis, dispersed, in about six weeks, the subcutaneous effusions, and rendered the breathing easier. His appetite returned, and he again left us.

On the 16th of January, 1819, he was once more brought to the hospital. His breathing was now very distressing, especially when he attempted to lie on his back; he therefore lay on his face, but then felt a " *beating in his throat*," at the top of the sternum.

The subcutaneous infiltrations were greater than ever. He was moreover affected with cough, diarrhœa, and acute pain in the epigastrium. The heart still gave a strong impulsion to the cylinder. Local bleeding, sinapisms, digitalis, and other means, afforded no relief, and the orthopnœa daily increased.

On the 4th of February, he threw up, almost without effort, a great quantity of vermillion blood, frothy, and mixed with sputa. The chest sounded well every where, on percussion; but the breathing could not be heard through the cylinder, opposite to the inferior portion of the right lung: and throughout the whole thorax a mucous rattling, or wheezing, could be heard, but more strongly in the right side. The words "*hæmoptysic engorgement*" were added to the former diagnosis on the books, and a large bleeding was prescribed.

"5th. In great distress; orthopnœa considerable; face somewhat shrunk; the blood brought up by coughing had lost its vermillion colour, and was less copious—same phenomena by the cylinder. The symptoms all went on increasing, and he expired on the 8th, after a long and agonizing struggle.

"*Dissection.* The brain and its meninges presented nothing remarkable. The pericardium contained about an ounce of serosity. The heart was nearly triple its natural size; it presented, on its surface, several patches of a mother-of-pearl appearance, irregular at their circumference, and about half the size of the palm of the hand. The right ventricle contained a polypus concretion partly organized, and extending into the corresponding auricle. The cavity of this ventricle somewhat dilated, and its parietes rather thickened. The right auricle offered nothing particular besides the polypus above mentioned. The parietes of the left ventricle were thickened to from nine to eleven lines, and of remarkable density. The cavity about twice its natural size. The mitral valve exhibited several cartilaginous patches extremely hard, developed in its substance. The arch of the aorta was somewhat aneurismal, and lined with cartilaginous and osseous incrustations. The right pleura contained about six ounces of fluid; and the lung of this side presented, towards its base, a zone, two or three inches in breadth, traversing the whole of its substance, exactly defined, and changing abruptly into sound and crepitous pulmonary tissue, from which it was easily distinguishable by a density equal to that of liver—a black red colour, and a granular surface when cut into. Its substance considerably resembled that of the corpus cavernosum penis. Three or four other engorgements of this kind, but of very small volume, were discovered in the same lung. The left lung contained, towards its base posteriorly, two or three small, but well defined engorgements, similar to those in the right side. The liver was smaller than natural, but studded with little yellow bodies, of a tubercular nature." *Vol. II.* p. 54-62.

Here we must close the pathology of the lungs and their coverings, and proceed to that of the *heart.*

In this country, of all others, the diseases of the respiratory organs are peculiarly worthy of investigation; and we hope that we have contributed to extend our pathological knowledge of that important viscus, through the medium of the above analysis—an analysis which has cost us no trifling labour, but for which we shall think ourselves amply rewarded by the approbation of our professional brethren.

### Part II. *Pathology of the Heart.*

As the individual derangements of structure affecting the heart, appear to be better classed and distinguished by M. Laennec, than by any other author, we shall now endeavour to convey to the English reader a concentrated analysis of this gentleman's diagnostics and symptomatology of these melancholy and interesting affections, some of which diagnostics we have put to the test of experience, and can vouch for their general accuracy.

### 1. *Hypertrophia Simplex, or active Aneurism of the Heart, without Dilatation of its Cavities.*

This disease, which, however, is not very common, has entirely escaped the notice of Corvisart. It is an augmentation and condensation of the muscular structure, and consequently of the ventricular parietes of the heart, not only without a corresponding increase of capacity in the cavities; but more commonly with a diminution of their volume. It may affect one or both ventricles, but rarely the auricles.

When the disease is seated in the left side of the heart, its parietes sometimes become more than an inch in thickness, at the base of the ventricle, which is double what they should be. There is a corresponding increase of substance in the carneæ columnæ of the valves, and in the septum ventriculorum. The muscular substance of the heart is much more dense, and of a deeper red colour than natural; while the cavity of the ventricle appears to have lost in capacity what the parietes have gained in thickness. The anatomical character of *hypertrophia sine dilatatione*, in the right side of the heart, does not differ materially from the above.

*Signs of Hypertrophia of the left Ventricle.* In addition to the general symptoms of diseases of the heart, which need not be described, may be put down, as nosological marks, which, however, are far from infallible, a strong and full pulse; strong pulsations of the heart, in the left side, equally felt by the patient himself, and by the hand of the practitioner; a want of sound on percussion of the cardiac region. Auscultation, however, by means of the stethescope invented by Laennec, affords more certain data for diagnosis. When the cylinder is applied between the fifth and sixth rib, the contraction of the left ventricle will communicate a very strong impulse, accompanied by a sound much more dull than in a healthy state of the parts. The contraction of the auricles is very short, and little sonorous. The beating of the heart can only be heard over a very small space. In this affection, more than in any other, the patient experiences a constant sense of the heart's action; but he is not subject to violent palpitations, excepting when under the influence of strong moral or physical agents. Neither is there much intermission, or other irregularity of the pulse.

*Signs of Hypertrophia of the right Ventricle.* According to M. Corvisart, these differ from those attending hypertrophia of the left ventricle only in being accompanied by a greater tightness of the chest in respiration, and a deeper colour of the face. Lancisi put down pulsation of the external jugular veins, as a sign of active aneurism of the right ventricle of the heart; but Corvisart rejects this diagnostic, because he saw it in cases of dilatation of the left ventricle. Laennec, on the other hand, observed this phenomenon in every case of considerable hypertrophia of the right ventricle. We have had an opportunity of verifying this statement of Laennec in a recent case. When the contractions of the heart are investigated by the cylinder, the same phenomena are presented as in the affection of the left ventricle, except that the *impulse* is stronger at the lower part of the sternum than opposite the apex of the heart, between the fifth and seventh ribs. Hypertrophia simplex of the *right* is still less common than hypertrophia of the *left* ventricle. In simultaneous hypertrophia simplex of both ventricles there is a combination of the symptoms above enumerated.

2. *Dilatation of the Ventricles of the Heart.* The *passive* aneurism of Corvisart presents the following pathological characters, anatomically examined. The cavities of the ventricles are enlarged, while their parietes are attenuated, and the muscular substance, in general, softened in consistence. The parietes of the left ventricle, in particular, are occasionally not more than a couple of lines in thickness, the muscular substance being sometimes of a violet hue, at others, pale or yellow. The carneæ columnæ, in these cases, appear more than naturally separated from each other, and the septum ventriculorum thinned, like the parietes. This simple dilatation may affect only one ventricle, but more commonly it extends to both; which is not the case in the opposite condition, namely, in hypertrophia simplex.

*Signs of Dilatation of the left Ventricle.* These, according to Corvisart, are, "soft and weak pulse; slight palpitations, as if a soft body raised the ribs, but did not come against their internal surface with a lively stroke; an absence of sound, on percussion, over a considerable extent of the cardiac region." But these signs are all fallacious. In general, the heart's action, in this complaint, cannot be felt at all, and the pulse cannot be depended on. The cardiac region will also sometimes sound well, when there is dilatation of the left ventricle to a great degree. The only certain diagnostic, is a clear sound transmitted through the stethescope, at each contraction of the heart, when the instrument is applied opposite the apex of this organ.* The degree of clearness in the sound, and the extent over which it is heard, are proportioned to the degree of dilatation. Thus, when the sound of the ventricular contraction is as clear as that of the auricle, and, at the same time, the action of the heart can be heard, by means of the cylinder, in various parts of the chest, and even in the back, the degree of dilatation is extreme.

*Signs of Dilatation of the right Ventricle.* According to Corvisart, these are nearly the same as those attending dilatation of the left, as far as regards the pulse, and tangible action of the heart. He does not, as was before mentioned, attach much importance to the jugular pulsation noticed by Lancisi. The more unequivocal phenomena, according to this veteran physician, are the greater dyspnœa than when the left ventricle is affected, the greater disposition to serous effusions, the more frequent occurrence of hæmoptysis, and a deeper livid tint of the face, sometimes amounting to a violet hue. Laennec has observed an *habitual turgidness* of the external jugular veins, without sensible pulsation, to be a very constant attendant on passive dilatation of the right ventricle. The absence of sound, on percussion, this accurate observer has found to be a very equivocal symptom, and in this light it has appeared to us in practice. Although, in general, the face exhibits a considerable degree of livor in this disease, yet there are many instances where it is extremely pale and leuco-phlegmatic; a remarkable case of which, lately fell under our own observation. It is also consonant with experience, that *active dilatation* of the right ventricle, that is, dilatation with hypertrophia, presently to be described, is more frequently accompanied by lividity of face and extremities, breathlessness, discharges of blood from the lungs, and serous effusions, than the passive dilatation now under consideration. The most certain pathognomonic sign of ventricular dilatation of the right side, according to Laennec, is the clear and distinct sound or noise produced by the heart's action, when explored by the stethescope, applied to the lower part of the sternum, or between the ribs of the left side. The truth of this we have lately had an opportunity of witnessing in two cases.

3. *Dilatation of the Ventricles, with Hypertrophia, or thickening of their Parietes.* The combination of these two affections is extremely common, infinitely more so, indeed, than simple dilatation without hypertrophia or increase of substance, the complaint investigated in the preceding section. This is the active aneurism of Corvisart, and may take place in one or in both ventricles at the same time. It is in this last case that the heart is sometimes found to have acquired a prodigious volume, twice, thrice, or even four times its natural size. With this increase of size in the muscular

* See Laennec's Work on Auscultation, vol. ii. p. 268.

structure of the heart, there is generally a proportionate increase of density.

The signs of this disease are a combination of those attending simple dilatation, and simple hypertrophia of the ventricles: thus the ventricular contractions will produce, through the stethescope, a strong impulsion, and a well marked noise, the auricular contractions being quite sonorous. The cardiac pulsations are heard over a great extent of the thorax, sometimes, especially in thin subjects and children, in every part of the chest.

In this active enlargement of the heart, its pulsation can not only be distinctly felt, and generally seen, in the cardiac region, but the head, the limbs, and even the clothes, will be observed to vibrate in consequence. The carotids, radials, and other tangible arteries, will also, in general, give evidence of this morbid force and action of the central organ of the circulation. If the hand be pressed against the ribs of the left side, the heart, according to the expression of Corvisart, "seems to be irritated thereby, and to react more strongly." "Cet organe semble s'irriter contre la pression et reagir plus fortement." The pulse in this, as in most other diseases, is very fallacious. Even in this state, and violent action of the heart, the pulse at the wrist will feel small, feeble, and regular. The cylinder applied alternately to the left side, and to the lower part of the sternum, will give the indications whether one or both ventricles are in a state of active dilatation. As it is in these cases, that the heart acquires the largest possible size, so it is here that we most frequently observe the absence of healthy and natural sound for a greater or less extent, on striking the cardiac region.

But it must never be forgotten, that, of all diseases, those of the heart are most difficult to be ascertained, by any one set of symptoms or mean of diagnosis. The whole history of the patient's complaint should be accurately investigated, and then percussion and auscultation employed in aid of the general phenomena; for it ought to be borne in mind, that active and passive dilatations of the heart are, after all, but *relative disproportions* between the circulating and other organs of the body, or between the different parts of the heart itself, and that an augmentation in the size of this organ, which, in a person of narrow chest and weak lungs, becomes the cause of perpetual sufferings, would, in a person whose chest was capacious, lungs sound, and capillary system of firm texture, occasion no inconvenience at all.* In short, there are few people whose hearts are in perfect and healthy proportion to the other organs and systems of the body, and this disproportion is the source of innumerable derangements of function and structure through life. It is, in general, much more advantageous to the constitution that the heart should be below than beyond the natural size, though a considerable degree of enlargement is not always incompatible with comfortable, if not robust health, and even long

* Vide Laennec, vol. ii. p. 275.

life, where there is no defect in any other organ or part of the body.

It is evidently of the greatest consequence, however, to discover this disproportion between the heart and other organs, before it begins to produce any serious inconvenience; for it is at this period that we can put in force certain preventive checks to future sufferings. One of the great advantages of auscultation is the early discovery of this tendency to enlargement of the heart.

The heart is subject to a great many other disorders of structure than are here enumerated, such as a fatty degeneration, a flabby, and, as it were, rotten state of the muscular substance, an induration of the same, and an atrophy, or wasting of the organ; but the symptoms attending these states are not yet sufficiently ascertained, and therefore their diagnosis must depend on the accumulated observations of future pathologists.

4. *Valvular Disorganizations.* There is a melancholy and distressing class of complaints, to which the heart is subject, denominated *ossification* of its valvular apparatus. It is curious that this species of induration is almost exclusively confined to the *left* side of the heart and the *arterial* system thereunto belonging, while the right side of the heart and veins, in other words, the system of *black blood*, is nearly or altogether exempt from such disorganization. Two inconveniences naturally result from induration, or, as it is called, ossification of the mitral valve in the heart, and semi-lunar valves of the aorta, the general seats of this lesion. In the first place, by their thickening and immobility, they obstruct the free passage of the blood from the auricle into the ventricle, and from the ventricle into the aorta. In the second place, by their immobility, they cease to perform the office of valves, and consequently, when the ventricle *contracts*, the mitral valve suffers a part of the blood to regurgitate back into the auricle; and when the ventricle *dilates*, the semi-lunar valves suffer a portion of blood to return from the aorta back into the heart. The derangement which the circulation must experience from this state of things, when the auriculo-ventricular, and the ventriculo-aortic openings have become considerably contracted or obstructed, may be readily imagined. The diagnostic symptom attending the ossifications in question, is difficult to describe, but extremely easy to recognize, after having been once observed. It is a murmuring noise or tremor, which has been not inaptly compared to the purring of a cat, that may be distinguished by the hand applied to the region of the heart, and in some measure heard, while the hand is placed on the ribs, but not unless the hand is there. When the stethescope, however, is used, the purring murmur is heard distinctly by the ear alone.

The constitutional symptoms attending this class of disorganizations, are those described as attending diseases of the heart, in general, and need not be recapitulated. The same remark applies to the treatment.

There is sometimes a difficulty in distinguishing organic diseases of the heart from functional disturbances occasioned by nervous irritability. It is well known that students in medicine very often fancy themselves to be affected with cardiac diseases, after hearing a lecture, and particularly a clinical lecture on this class of complaints. Many of Corvisart's pupils pined—some of them for years—in misery, supposing themselves to be victims to this dreaded malady, after walking the hospitals with that celebrated physician, and having their attention directed to diseases of the heart by a man who so long and so successfully cultivated this branch of pathology. Literary men, of all descriptions, from the increased sensibility of their nervous systems, resulting from sedentary habits and intellectual cultivation, are very liable to various affections of the vascular system, which imitate organic diseases of the heart or great vessels, causing considerable alarm and disquietude. Among these, palpitations in the chest, and pulsations at the pit of the stomach, or lower down in the belly, are the sources of great anxiety. Although these symptoms are not to be despised, or carelessly examined into by the medical attendant, they are, for the most part, attributable to nervous irritability, and consequently are not, in themselves, dangerous. But it should be borne in mind that disordered *function* may, and too often does, in time, produce alteration of structure; and then we are awakened, when it is too late, to the real state of the case.

We have thus endeavoured to give as complete a view of M. Laennec's work as could reasonably be expected within the compass of an analytical article. But a considerable mass of important matter was still left untouched. We are happy to say, however, that a translation of the work is in the press, and we need hardly state, that we consider it as one of the most valuable additions to our pathological knowledge of thoracic affections which the present age has produced. We are sincerely of opinion, that those who neglect to possess themselves of the work, either in the original or translation, inflict a deep wound on their best interests. To the enlightened author, of whom France may be justly proud, the thanks of Europe are due.*

---

* The Editor having procured some cylinders from Paris, has engaged a workman in London to make them for any gentleman in town or country, who may wish to have one. They are constructed of box or other close wood, by Mr. Alnutt, in Piccadilly, at four shillings each. Models of the instrument may be seen at the Editor's residence at any time.

